# Safety and Efficacy of an Implantable Cardioverter Defibrillator (ICD) in the Detection and Prevention of Cardiac Arrhythmia - A Systematic Review

**DOI:** 10.7759/cureus.48471

**Published:** 2023-11-07

**Authors:** Hadrian Hoang-Vu Tran, Mingma L Sherpa, Nilasma Shrestha, Niriksha Ravi, Silpa Choday, Vivig Shantha Kumar, Anil KC, Anusha Parisapogu, Blessing T Ojinna, Lubna Mohammed

**Affiliations:** 1 Internal Medicine, California Institute of Behavioral Neurosciences & Psychology, Fairfield, USA; 2 Neurology, California Institute of Behavioral Neurosciences & Psychology, Fairfield, USA; 3 Medicine, California Institute of Behavioral Neurosciences & Psychology, California, USA; 4 Internal Medicine and Neurology, California Institute of Behavioral Neurosciences & Psychology, Fairfield, USA; 5 Internal Medicine and Family Medicine, California Institute of Behavioral Neurosciences & Psychology, Fairfield, USA; 6 Infectious Diseases, Mayo Clinic, Rochester, USA; 7 Internal Medicine and Pediatrics, California Institute of Behavioral Neurosciences & Psychology, Fairfield, USA; 8 Medicine, University of Nigeria Nsukka, Enugu, NGA

**Keywords:** life-threatening arrhythmia, intervention cardiology, cardiology devices, implantable cardiac defibrillator (icd), safety and efficacy

## Abstract

Implantable cardioverter defibrillators (ICD) have been recommended as an effective therapy in treating sudden cardiac deaths. This study evaluates the safety and efficacies of ICDs in detecting arrhythmias. Different ICDs, such as the transvenous cardioverter defibrillator (TV-ICD) and the subcutaneous implantable cardioverter defibrillator (S-ICD), are used. This systematic review identified Embase, PubMed, Medical Literature Analysis and Retrieval System Online (MEDLINE), and Web of Science as the primary electronic databases for research. Supplementation of the available articles for the review was done using Google Scholar. The population, exposure, control, outcome, and studies (PECOS) criteria were used in this study. The quality of the included studies was assessed using the Critical Appraisal Skills Program (CASP) standard checklist. Preferred Reporting Items for Systematic Reviews and Meta-Analyses (PRISMA) guidelines were used in this systematic review.

Two researchers conducted the extraction of data. A pre-designed Excel worksheet (Microsoft, Redmond, Washington) was used in the recording of extracted data. Eight studies were identified for use in this systematic review. Safety of the ICDs was observed with the minimum number of reported inappropriate shocks. Studies conducted identified that women had a lower number of incidences when a long detection setting by sex was conducted. Strategic programming of ICDs was noted as effective in lowering the levels of mortality. Studies claimed that the reduction of inappropriate shocks were important in the reduction of myocardial damage, which resulted in the mortality rate among the patients decreasing. Having high cutoff rates and long intervals for detection in ICD programming was noted to help in reducing ICD therapy intervention among patients. Differences among the male and female populations were inconsequential in the efficacy and safety of ICDs. Their effectiveness in sensitivity, pacing success, and defibrillation success were high and very significant. ICDs were safe in their use in the detection of arrhythmias.

## Introduction and background

Implantable cardioverter defibrillators (ICD) have been recommended as an effective therapy in treating sudden cardiac deaths [1]. This has been critical in patients with primary cardiac electrical syndromes or those with structural heart disease. Through the years, there have been technological advancements in ICDs, which have bettered their use and increased the scope of their therapies [2]. ICDs have evolved from treating ventricular tachycardia or fibrillation to being part of resynchronization therapy, supporting bradycardia-pacing, and availing complex discrimination algorithms [1]. Different types of ICDs are used, such as the transvenous implantable cardioverter defibrillator (TV-ICD) and the subcutaneous implantable cardioverter defibrillator (S-ICD) [3]. The S-ICD comprises a titanium case covering the subcutaneous pulse generator and a subcutaneous lead. Distal and proximal sensing electrodes, separated by a three-inch shock coil, are the lead's contents [4]. The S-ICD can deliver post-shock transthoracic pacing for up to 30 seconds [5]. However, it is limited to bradycardia or anti-tachycardia pacing (ATP). Transvenous implantable cardioverter defibrillators have been cited for complications despite their ability to increase survival chances for people with a risk for sudden cardiac deaths. For several decades, the transvenous lead placement had been the standard for cardiac sensing and defibrillation in ICDs [6]. The introduction of subcutaneous ICDs was done to mitigate the risks from TV-ICDs. Studies indicated risks such as cardiac perforations and pneumothorax associated with lead insertions. S-ICDs have extra-thoracic placements, which remove the necessity for vasculature or getting into the heart [7].

Preventing sudden cardiac deaths, whether primary or secondary, has used ICDs as the standard therapy for patients with a high risk of ventricular tachyarrhythmia (VT/ VF) [8]. Studies conducted identified that increasing the time for detecting VT/ VF and having cutoff rates set high helped reduce ICD therapies deemed unnecessary. ICDs are generally small battery-powered devices that detect arrhythmias and help stop them [9]. Continuous monitoring of heartbeats is done by the ICDs, which provide electric shocks in cases of arrhythmias to return them to regular. They are provided in instances where patients have symptoms of sustained ventricular tachycardia. Studies have also observed that ICDs are recommended in cases of enlarged heart muscles or histories of a weakened heart due to coronary artery diseases [10]. ICDs could be programmed to either low energy pacing or higher energy shock, depending on the heartbeat problem. Low energy pacing deals with mild heartbeat changes and produces a painless fluttering sensation. However, higher energy shocks have shocks that may be painful due to their high energy. Such shocks deal with serious heart rhythm problems [10]. There has also been the use of dual-chamber ICDs [11, 12].

ICDs in the detection of tachycardia or fibrillation use the mean ventricular rate. The mean ventricular rate is the number of short consecutive intervals. An ICD detects arrhythmias when the number exceeds what was programmed [11]. Such programming helps reduce the risk of ventricular fibrillations due to ventricular under-sensing and helps limit inappropriate therapies due to sinus rhythms, which may cause premature ventricular complexes [11]. Benefits that may arise from using ICDs can be downplayed due to adverse events caused by the delivery of shocks [13]. Some studies identified that some ICDs could increase mortality for being pro-arrhythmic despite delivering appropriate shocks [14]. Increased detection time of the ICDs appeared to be very beneficial [13].

## Review

Research Aims

This study will seek to investigate the safety and efficacy of ICDs in the detection of cardiac arrhythmias.

Methods

Study Design

Preferred Reporting Items for Systematic Reviews and Meta-Analyses (PRISMA) guidelines were used in this systematic review. The preparation for this systematic review also included the use of PRISMA extensions published in the Cochrane Handbook for Systematic Reviews and Extensions [15].

Search Strategy

This systematic review identified Embase, PubMed, Medical Literature Analysis and Retrieval System Online (MEDLINE), and Web of Science as the primary electronic databases for research. Supplementation of the available articles for the review was done using Google Scholar. The search strategy used keywords, keyword combinations, Medical Subject Headings (MeSH) terms, field tags, Boolean operators "AND" and "OR," and truncations. Search strings were built from these elements to ensure an accurate acquisition of the best articles. Table 1 shows the MeSH terms and keywords that were used in the search of appropriate articles. Identified articles were sought to get the most relevant for this systematic review.

**Table 1:** Search strategy used in the study MeSH - medical subject headings; ICD - implantable cardioverter defibrillators

Database	Search strategy
PubMed	"Safety"[MeSH Terms] OR safety[Text Word], efficacy[All Fields], "defibrillators, implantable"[MeSH Terms], "defibrillators, implantable/adverse effects"[Mesh], "arrhythmias, cardiac/diagnosis"[Mesh]
Medical Literature Analysis and Retrieval System Online (MEDLINE)	"Defibrillators, implantable/standards"[Mesh], implantable cardioverter defibrillator[Text Word], "arrhythmias, cardiac/therapy"[Mesh], "defibrillators, implantable/statistics and numerical data"[Mesh]
Web of Science	Implantable cardioverter defibrillators, cardiac arrhythmias, ICD, safety and efficacy
Embase	Safety and efficacy, implantable cardioverter defibrillators, ICD, cardiac arrhythmia

Eligibility Criteria

The researchers selected eligibility guidelines for the selection of studies to be included in this systematic review. The population, exposure, control, outcome, and studies (PECOS) criteria were used in this study. The population in the included studies had to be patients having implantable cardioverters or defibrillators. Exposure considered for inclusion was the use of implantable cardioverter defibrillators. This systematic review had no specific comparator for the control. The safety and efficacy of the ICDs were the outcomes prioritized for this study. Study designs considered for this systematic review were clinical trials and randomized clinical trials (RCT). Consideration was also done for articles with different study designs but had very relevant material. Only English-published articles or those translated were considered for inclusion.

Quality Assessment

The quality of the included studies was assessed using the Critical Appraisal Skills Program (CASP) standard checklist. Quality assessment is conducted using four sections. Section I utilizes three questions to validate the study designs of the included studies. The methodological soundness of the included studies was assessed using three questions. The validity of the results obtained was appraised using three questions, while the applicability of the results was assessed using two questions. "Yes", "No", and "Can't Tell" were the responses used for assessment. These responses were abbreviated to "Y", "N", and "CT". Responses from the checklist were used in ranking the studies for quality appraisal, where the highest score was eleven. High-quality studies had a score ranging from eight to ten. Scores of six and seven were of moderate quality, while those with a score of less than five were of low quality. Table 2 below shows the questions used in the checklist.

**Table 2:** CASP standard checklist used in this systematic review CASP - Critical Appraisal Skills Program

Study evaluation criteria
C1. Was the research question from the study focused?
C2. Was randomization of the participants towards the interventions done?
C3. In conclusion, was there accountability of the participants?
C4. Was blinding done for the following: - The participants? - The investigators? - Results analyzers?
C5. Were the study groups similar at the start of the trial?
C6. Was there a similarity in the level of care among the study groups and the participants?
C7. Was a comprehensive report on the effects of the interventions done?
C8. Was there a report on the precision of the effect of treatment or the estimate of the intervention?
C9. Were the safety and efficacies of the implantable cardioverter defibrillators identified?
C10. Was there compatibility between the population and the results?
C11. Was there a benefit in the application of implantable cardioverter defibrillators compared to those without?

Data Extraction

Two researchers conducted the extraction of data. A pre-designed Excel worksheet (Microsoft, Redmond, Washington) was used in the recording of extracted data. Information on the authors, year of publication, demographic, aims, outcomes, and the results of the included studies was extracted. Engagement between the two researchers was constant to ensure the results' congruence. A third party quelled disputes that arose.

Results Analysis

This systematic review made use of only one form of investigative analysis. The method used was a qualitative assessment and a systematic review. Literal analysis was also conducted from the included studies.

Results

Study Selection

**Figure 1:**
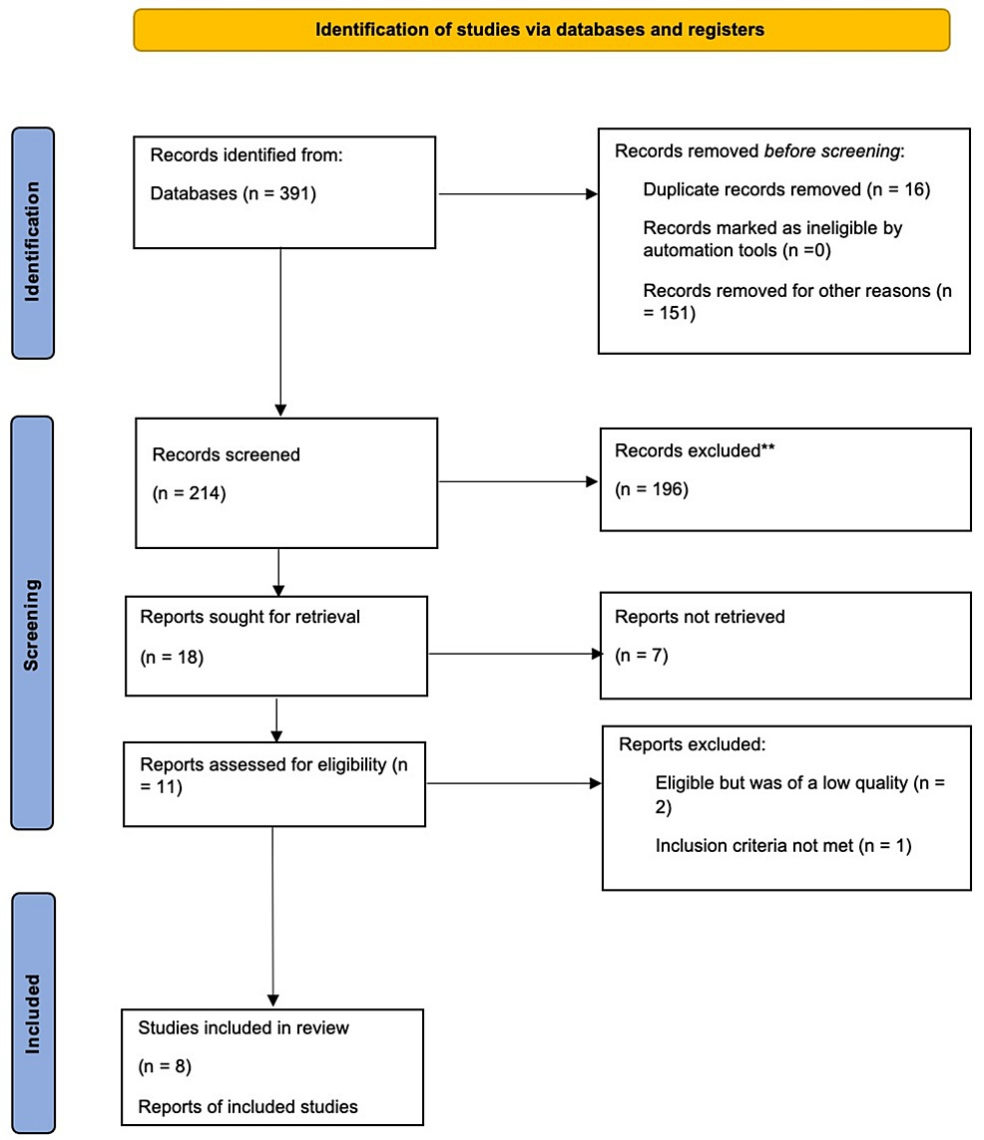
PRISMA flow diagram of the inclusion and exclusion process of the studies in the systematic review n - number of studies; PRISMA - Preferred Reporting Items for Systematic Reviews and Meta-Analyses

Following a general search of the databases Embase, PubMed, MEDLINE, and Web of Science, a total of 391 studies were identified. Of these, sixteen articles were identified as duplicates, while 151 were irrelevant and were subsequently removed, remaining with 214 studies. Screening of the remaining articles assessed their titles, abstracts, and whether they were full texts. A total of 196 studies were excluded, with 18 studies remaining. Seven studies were excluded from the remaining studies as they were irretrievable. Eleven studies were assessed for their eligibility; two were excluded for having low quality, while one was excluded for not meeting the criteria for inclusion of studies. Eight studies were identified for use in this systematic review. Figure 1 below shows the process of inclusion and exclusion [1, 8, 16-21].

Quality Analysis

Quality analysis of this systematic review was conducted using the CASP standard checklist. From the analysis, two studies [8, 19] were of the best quality, scoring eleven. The lowest score was eight, identified in four studies [1, 17, 18, 20]. The rest of the studies [16, 21] scored nine. The included studies had relevant and sufficient data for this study. Table 3 shows the scores from the CASP tool analysis [1, 8, 16-21].

**Table 3:** Scores obtained from the CASP tool for analysis CASP - Critical Appraisal Skills Program; CT - Can't tell; N - No; Y - Yes

Author	C1	C2	C3	C4	C5	C6	C7	C8	C9	C10	C11
Adduci et al. (2018) [1]	Y	CT	Y	CT	Y	Y	Y	Y	Y	Y	Y
Gasparini et al. (2020) [8]	Y	Y	Y	Y	Y	Y	Y	Y	Y	Y	Y
Swerdlow et al. (2021) [16]	Y	N	Y	Y	Y	Y	Y	Y	Y	Y	Y
Crozier et al. (2020) [17]	Y	N	Y	N	Y	Y	Y	Y	Y	Y	Y
Sasaki et al. (2018) [18]	Y	Y	Y	Y	Y	Y	N	N	Y	Y	Y
Buber et al. (2014) [19]	Y	Y	Y	Y	Y	Y	Y	Y	Y	Y	Y
Hayashi et al. (2017) [20]	Y	CT	Y	CT	Y	Y	Y	Y	Y	Y	Y
Oginosawa et al. (2017) [21]	Y	CT	Y	Y	Y	Y	Y	Y	Y	Y	Y

Study Characteristics

The study characteristics were identified and extracted on a pre-designed Excel worksheet. Table 4 shows the study characteristics extracted [1, 8, 16-21].

**Table 4:** Data extracted from included studies EMI - electromagnetic interference; VT - ventricular tachycardia; SVT - supraventricular tachycardia; VF - ventricular fibrillation; VFL - ventricular flutter; BMI - body mass index; LVEF - left ventricular ejection fraction; HR - hazard ratio; CI - confidence interval; CRTD - cardiac resynchronization therapy with defibrillator; NYHA - New York Heart Association; LVDD - left ventricular end-diastolic diameter; AFL - atrial flutter; ATP - appropriate delivered therapy; VA - ventricular tachyarrhythmia; NID - number of intervals needed to detect VT/VF; SST - smart-shock technology; ICD - implantable cardioverter defibrillator; EGM - electrogram; S-ICD - subcutaneous implantable cardioverter defibrillator; TV-ICD - subcutaneous versus transvenous implantable defibrillator; EV-ICD - extravascular implantable cardioverter defibrillator

Author	Study design	Demography	Intervention	Control	Aims	Outcomes	Results
Adduci et al. (2018) [1]	Review	Analysis of 882 patients. Follow-up = 651 ± 345 days. 72% were male. 29% had previous myocardial infarctions.	S-ICD : n = 985	TV-ICD: n = 1637	The study aimed to highlight the safety and efficacy data of S-ICD in its role in clinical practice	Safety and efficacies	Infection rate: 2% vs 1.6%. Need for explant: 1.7% vs >50%. Endocarditis: 0% vs 22 - 54%. Implant complications: Hematoma: 4% vs 0.86-2.4% Device erosion: 1.2-3% vs 1.5% Inappropriate shocks: 1.6% vs 7-10%(first year) 18 years (5 year follow up) Electrode dislodgement: 0.6% vs 1.8% S-ICD efficacy: Sensitivity: 100% Shock efficacy: 98%
Gasparini et al. (2020)[8]	Randomized, controlled, single-blind and multicenter study	Mean age: 65 (12) vs 65 (11) Permanent atrial fibrillation: 26 (9%) vs 186 (12%) Secondary prevention: 72 (24%) vs 405 (25%) VF/V flutter: 35 (12%) vs 139 (9%) NYHA class III or IV: 166 (55%) vs 724 (46%) LVEF (%): 31 (11) vs 30 (9) LVDD (mm): 60 (8) vs 64 (9) Beta-blockers: 245 (81%) vs 1299 (81%) Single-chamber ICD: 79 (26%) vs 466 (29%) Dual-chamber ICD: 69 (23%) vs 513 (32%) Triple-chamber ICD: 156 (51%) vs 620 (39%)			This study sought to compare and analyze long-detection programming on time to first device therapy between men and women.	Inappropriate shocks, sensitivity, ATP	ARRHYTHMIA AND CERTAIN DEATH: (Appropriate therapies 95% CI, HR) Shock: 0.46 (0.17 to 1.21) vs 0.83 (0.60 to 1.15) ATP: 0.36 (0.15 to 0.87) vs 0.61 (0.46 to 0.81) Inappropriate therapies: Shock: 0.40 (0.08 to 2.09) vs 0.58 (0.34 to 1.01) ATP: 0.68 (0.11 to 4.07) vs 0.63 (0.37 to 1.04) Sensitivity Analysis: NID 18/24: 151 (50%) vs 152 (50%) NID 30/40: 153 (50%) vs 151 (50%)
Swerdlow et al. (2021) [16]	Clinical trial	Mean age: 53.0 ± 13.1 BMI: 31.1 ± 4.5 New York Heart Association Functional Class I: 4 patients II: 8 III: 7 IV: 1 Cardiomyopathy: 16 Ischemic: 6 Non-ischemic: 6 Left-ventricular ejection fraction (%): Left-ventricular ejection fraction (%): 43.1 ± 18.5	EV -ICD: n = 20		The study reported on the performance of sensing and detection of arrhythmia of extravascular ICDs in a human pilot study.	Under-sensing and over-sensing: Noise discrimination, effects of source, posture, and lead maturation.	Monomorphic VT: 1 episode misclassified by Wavelet, one misclassified by feature match, both monitored VT supraventricular tachycardia: 116 episodes discriminated by Wavelet, three by Feature Match Ventricular Over-sensing: 2 episodes discriminated by Sensed EMI, one by morphology noise, one by sensed noise discriminator
Crozier et al. (2020)[17]	Prospective, nonrandomized, chronic, first-in-human study	Patients had to be using ICDs and had class I or II indications. Exclusion occurred in cases of indication for bradycardia pacing or cardiac resynchronization therapy. 81% were men.	EV-ICD: n = 26	_	This study evaluated the Safety and performance of EV-ICD.	Safety and performance of EV-ICD	Median defibrillation threshold: 15j. Ddefibrillation success: 90% ( ≥10-J safety margin). Mean R-wave amplitude: 3.4 ± 2.0 mV. Mean ventricular fibrillation amplitude: 2.8 ± 1.7 mV. Pacing success: 95% (≤10 V)
Sasaki et al. (2018)[18]	Retrospective observational study	Mean age: 60 (44–67) vs 69 (58–79) BMI: 24.4 (20.9–27.0) vs 23.5 (22.1–26.6) Atrial fibrillation: 4(7) vs 8(17) LVEF (%): 58 (39–66) vs 35 (28–57)	S-ICD: n = 60	TV-ICD: n = 47	This study sought to report on the experiences of patients treated with S-ICD in Japanese institutions.	Safety and Efficacy	Defibrillation test: 56 patients (93%). Ventricular fibrillation (VF): single 65-J shock. Shock therapy: 13.4 s (IQR, 12.1–14.9s). Median post-shock impedance: 64Ω (IQR, 58–77Ω). Over-sensing of myopotential: n = 3. T-wave: n = 1. Supraventricular tachycardia detection: n = 1. Inappropriate shocks: 5% - 15%
Buber et al. (2014)[19]	Retrospective analysis	Mean age: 64(14) vs 65(11) Male: 124(89) vs 144(90) Hypertension: 81 (58) vs 94 (59) Atrial fibrillation: 12 (9) vs 15 (24) Antiarrhythmic treatment: 24 (18) vs 21 (13)Beta-blocker treatment: 104 (74) vs 145 (91) Ejection fraction: 256 vs 266 Single chamber: 35 (25) vs 56 (35) Dual chamber: 48 (34) vs 61 (38) CRT-D: 41 (57) vs 43 (27) NYHA class: NYHA 1 + 2 : 69 (49) vs 83 (52) NYHA 3 + 4: 71 (51) vs 77 (48)	Physician tailored programming: n = 140; Single chamber (n = 35)/ Dual chamber (n = 48)/ CRT-D (n = 57)	Strategic programming: n = 160; Single chamber (n = 56)/ Dual chamber (n = 61)/ CRT-D (n = 43)	This study sought to evaluate the effectiveness and safety outcomes of strategic programming, which reduced the shock delivery burden among patients implanted with ICDs for prevention compared to traditional physician preference programming.	The composite of death and appropriate shocks, actual syncope events, and untreated non-sustained VT events.	[Values (%)] Total events: 11 (31)/ 14 (29)/ 8 (14) vs 3 (5)/ 3 (5)/ 2 (5) SVT: 5 (14)/ 13 (27)/ 5 (9) vs 1 (2)/ 0/ 1(2) Sinus tachycardia: 4 (11)/ 0/ 1(2) vs 2 (4)/ 1 (2)/ 0 Abnormal sensing: 0/ 0/ 2(4) vs 0/ 1(2)/ 1(2) Unclassified: 1 (3)/ 1 (2)/ 0 vs 0/ 1(2)/ 0 (Multivariate analysis; HR; 95% CI) Strategic programming: 0.30; 0.17–0.62. Atrial fibrillation: 1.9; 1.05–3.2. Dual-chamber device: 1.2; 0.67–2.10. Beta-blocker treatment: 0.69; 0.33–1.09. Antiarrhythmic treatment: 0.62; 0.27–0.99. Ischemic heart failure: 1.1; 0.74–1.9
Hayashi et al. (2017)[20]	Retrospective Analysis	Mean age: 57 ± 13 Male: 58(89%) Single-chamber ICD: 7 (11 %) Dual-chamber ICD: 56 (86 %) CRTD: 2(3%) Brugada syndrome: 20 (31 %) Ischemic heart disease: 19 (29 % Dilated cardiomyopathy: 10 (16 %) [Conventional setting vs Strategic Setting] NYHA class I: 18 (29 %) vs 45 (69 %) Class II: 18 (29 %) vs 19 (30 %) Class III: 1 (1 %) vs 1 (1 %) Class IV: 1 (1 %) vs 0 Beta-blocker: 29 (45 %) vs 31 (48 %) LVEF: 46 ± 16 vs 47 ± 15 LVDD (mm): 53 ± 10 vs 54 ± 11 AF/AFL/AT: 10 (15 %) vs 13 (20 %)	ICD: conventional setting n = 65 vs Strategic setting n = 65	_	This study sought to evaluate ICD programming safety and Efficacy that were combined with long detection intervals having high-rate cutoffs for secondary prevention patients.		Overall (ATP + shock): 281 vs 33 ATP: 140 VS 13 Shock: 141 vs 20. Inappropriate delivered therapy overall: 56 vs 4. ATP: 40 vs 4. shock: 16 vs 0
Oginosawa et al. (2017)[21]	Prospective, multicenter, nonrandomized, observational trial	Mean age: 63.8±14.8 Men: 135 (74.2) Ischemic cardiomyopathy: 54 (29.7) Atrial fibrillation: 45 (24.7) Atrial tachycardia/flutter: 11 (6.0) NYHA I: 74 (40.9) NYHA II: 53 (29.3) NYHA III: 42 (23.2) NYHA IV: 12 (6.6) LVEF (%): 41.2±17.3	Conventional	SST	This study aimed to identify SST algorithms' benefits in ICDs or CRT-Ds.	The time to first inaccurate detection of ventricular tachycardia.	Inaccurate detection of ventricular tachyarrhythmia; Atrial fibrillation/flutter: 26(12) vs 23(14) Other SV tachyarrhythmia: 178 (84) vs 141 (86) T-wave over-sensing: 9 (5) vs 0

Discussion

Study Analysis

ICD innovation, as observed from studies conducted, was revolutionary in detecting arrhythmias and surgical implants, and preventing sudden cardiac deaths [1]. S-ICD implants used in trials from the EFFORTLESS registry were identified as having a sensitivity of 100% in detecting induced VF, while their shock efficacy was 98% [1]. Successful conversion of the S-ICDs among the patients was at a high of 98%. Conversion of spontaneous ventricular arrhythmia was identified at about 95.2% to 100% [1]. Such cases showcased the efficacy of ICDs in the detection of arrhythmias in a pursuit to mitigate them and avoid adverse effects. Studies identified that transient over-sensing of ICDs was a common phenomenon [16]. However, over-sensing that the S-ICDs sustained did not lead to inappropriate therapies or shocks [18]. Such actions were brought about by introducing discrimination features within the ICDs, which only caused inappropriate shocks in cases of displaced lead [16]. During stress testing, sustained over-sensing was observed in secondary vectors in high-amplitude myopotentials [16].

The studies' analysis identified that most of the patients and the users of ICDs were males [1, 8, 16-21]. Women were observed to have lower ICD therapies compared to men [8]. Therapies from ICDs were decreased by 69% (p=0.007) for women, and for men was 31% (p=0.006). Differences among the male and female populations were inconsequential in the efficacy and safety of ICDs. The use of ICDs in health safety was identified as high as they were indicated to have infection rates of 2% for S-ICDs and 1.6% for TV-ICDs [1]. Complications brought about by the implants were also minimal for the patients under study. Complications such as device erosion occurred at 1.2-3% for S-ICDs and 1.5% for TV-ICDs. Explants for ICDs were only high in TV-ICDs, which saw the need at > 50%. That was very high compared to S-ICDs, which ranged at 1.7% [1]. The lead component caused a high need for explants in TV-ICDs.

Strategic programming used in enhancing ICDs was reported to be safe and effective in detecting arrhythmias [19, 21]. Analysis of the studies identified that patients dreaded complications associated with inappropriate shocks as a safety concern [20]. Inappropriate shocks were causes of decreased quality of life, psychological issues, and risk increases in mortality [8, 19]. Improvements in ICDs using discrimination algorithms eliminated the risks of shock deliveries for supraventricular rhythms [19]. ICDs were nonetheless safe in their execution for the detection of arrhythmias. Using strategic programming and discrimination algorithms increased the effectiveness and the safety of the ICDs. The success in the efficacy of using ICDs was observed from the analysis of studies. Success in defibrillation was at 90% (≥10-J safety margin) and a pacing success of 95% (≤10 V) [17]. ICDs were majorly efficient in their detection of arrhythmias, having very low incidences of over-sensing, under-sensing, or inappropriate shock delivery. Their effectiveness in sensitivity, pacing success, and defibrillation success was high and very significant.

Safety

Safety of the ICDs was observed with the minimum number of reported inappropriate shocks [1]. Inappropriate shocks were mostly experienced among the subjects due to lead migration, supraventricular tachycardia (SVT), and T-wave over-sensing. However, to avoid inappropriate shocks, the selection of patients is an important feature that can be conducted using pre-implantation ECG screening. Such screening is boosted by exercise testing, which assesses vector eligibility among hypertrophic cardiomyopathy patients. Proper selection of patients that can have ICDs helped reduce inappropriate interventions. Studies conducted identified that women had a lower number of incidences when a long detection setting by sex was conducted [8]. The long detection had an actuarial freedom of one year from ICD therapies. There was reduction of first therapy risk among the patients that had the long detection setting when compared to the standard setting. This setting had the same results in both men and women. The reduction of antitachycardia pacing program (ATP)-delivered therapies helped reduce ICD therapy risk among patients. The sex of patients was identified as inconsequential on the effect of detection that were reported from the first therapy risk. Long detection helped in the reduction of inappropriate therapy risk all the more among women as compared to men.

S-ICDs were observed as having a higher level of safety as compared to TV-ICDs due to their avoidance of lead complications, which included venous obstruction and lead failure [18]. S-ICDs had very low cases of complications when used. Some of the complications that were identified as common included skin erosion and infections, which required the removal of the ICDs. S-ICDs were identified as having a limitation of having T-wave over-sensing. Such complications were, however, at low ranges. Inappropriate shocks among patients were caused by myopotential interference during daily life activities and over-sensing of the T-wave when at rest [18]. Studies reported that inappropriate shocks were majorly encountered in S-ICDs from one year onwards after implantation. The use of strategic programming in ICDs was reported as having the ability to reduce the risk of inappropriate shocks and mortality cases by very huge margins [19].

Under-utilization of ICDs was reported to occur due to the fear of complications such as inappropriate shocks [19]. Shock delivery was observed as being a traumatic event for the patients, whether appropriate or not. Such an event led to increased mortality, psychological issues, and a decrease in quality of life. Whenever inappropriate shocks were experienced, they increased the levels of trauma among the patients. However, the development of discrimination algorithms helped eliminate the delivery of shocks for supraventricular rhythms. Strategic programming of ICDs was noted as effective in lowering the levels of mortality. Studies claimed that the reduction of inappropriate shocks was important in the reduction of myocardial damage, which resulted in the mortality rate among the patients decreasing. Studies conducted reported that patients who had ICDs with strategic programming were able to tolerate VT events that were non-sustained.

Efficacy

Implantation of S-ICDs required defibrillation threshold testing (DFT), which assessed the shock efficacy and the sensitivity for ventricular arrhythmias [1]. DFT tests saw high detection sensitivity, which scaled up to 100% for VF, and a reported shock efficacy of 98%. This entailed the efficacy experiences for patients who used ICDs. Inspection of electrograms (EGMs) for VF was observed to have no effect in sensitivity due to sensing parameters reprogramming [16]. Virtual sensitivity for ICDs allows for the comparison of detection and sensing using different sensing parameters and episodes of VF that are the same. Studies observed that it was common for investigators to have sensitivity settings adjusted to ensure a two-fold detection margin of safety was achieved. The analysis of virtual sensitivity was identified as a recommendation for enhancing sensitivity reprogramming. It could be done due to under-sensing when spontaneous VF occurred or when over-sensing occurred at the baseline rhythm.

Extravascular (EV) ICDs were identified as being highly efficient in sensing, pacing, and acute defibrillation [17]. EV ICDs are known to provide pacing therapies and defibrillation from EV sub-sternal space. Limitations that are identified in transvenous defibrillators are avoided using the EV ICDs. The proximity of the EV ICD's lead to the heart enabled a requirement of lower energy for cardiac defibrillation. It also helped in the provision of asystole support pacing and ATP features. The termination of atrial fibrillation (AF) using S-ICDs was increased by the provision of higher current as compared to TV-ICDs across the left atrium during defibrillation [18]. The liberal use of ATP and long detection time approaches were identified as being effective for ICDs [19]. Having high cutoff rates and long intervals for detection in ICD programming was noted to help in reducing ICD therapy intervention among patients [20]. Using a new discrimination algorithm in some studies identified that inappropriate shocks were decreased effectively over a period of five years [21]. The computer modeling simulation that was used was applied in cases of 188 beats per minute and above. The inclusion of the smart shock technology algorithm with modern programming strategies was identified as reducing inappropriate shocks significantly. Inappropriate shock delivery by ICDs was reduced by the use of discrimination algorithms.

Limitations

This systematic review was limited during the identification of relevant articles for inclusion. The majority of the studies present ranged in publication age between 1994 - 2005. Recent studies were few and mainly focused on S-ICDs. This lacked in the diversification of assessing various types of ICDs.

## Conclusions

This systematic review identified the safety and efficacies that resulted from the use of implantable cardioverter defibrillators in the detection of arrhythmias. Efficacy was observed in their success in defibrillation, sensitivity, accurate detection, and delivery of appropriate shocks. ICDs had high success rates among the patients who were implanted. The analysis of virtual sensitivity was identified as a recommendation for enhancing sensitivity reprogramming. It could be done due to under-sensing when spontaneous VF occurred or when over-sensing occurred at the baseline rhythm. Strategic programming of algorithms used for the ICDs improved their effectiveness immensely. Having high cutoff rates and long intervals for detection in ICD programming was noted to help in reducing ICD therapy intervention among patients. Proper selection of patients that can have ICDs helped reduce inappropriate interventions. ICDs had low risks of complications among those who used them. This, therefore, meant that ICDs were safe in their use in detecting arrhythmias.
